# Factors Associated with Weight Change in Adults with Severe Mental Illness: Results from a Large Cross-Sectional Survey

**DOI:** 10.3390/nu17091423

**Published:** 2025-04-23

**Authors:** Gemma D. Traviss-Turner, Ellen Lee, Peter Pratt, Andrew J. Hill, Emily Peckham

**Affiliations:** 1 Leeds Institute of Health Sciences, University of Leeds, Clarendon Way, Leeds LS2 9JT, UK; a.j.hill@leeds.ac.uk; 2 School of Medicine and Public Health, University of Sheffield, Regent Court, 30 Regent St., Sheffield S1 4DA, UK; e.lee@sheffield.ac.uk; 3 National Speciality Advisor Mental Health Pharmacy, NHS England, Wellington House, London SE1 8UG, UK; peter.pratt@nhs.net; 4 School of Health Sciences, Bangor University, Gwynedd LL57 2DG, UK; e.peckham@bangor.ac.uk

**Keywords:** weight gain, obesity, antipsychotic medication, severe mental illness

## Abstract

***Background/objectives***: Individuals with severe mental illness (SMI) have a reduced life expectancy of 15–25 years. This is due to a number of modifiable and non-modifiable risk factors. Levels of overweight and obesity in this group are 1.8 times higher than in the general population and diet quality is poor. Excess weight is linked to a range of serious long-term physical and mental health conditions. This paper presents the findings of a large cross-sectional survey of adults living with SMI. The survey aimed to understand the current weight, weight gain and diet quality of this group and secondly, to explore the association between weight change, diet quality, antipsychotic medication and weight management. ***Methods***: Five hundred and twenty-nine participants (58% male, mean age 49.3) living with SMI completed the survey. ***Results***: Results showed 42% of the sample were living with obesity and almost half reported having gained 6 kg or more at least once in their adult life. Overall, 6% of the sample reported eating no fruit or vegetables and the same proportion had the highest consumption of carbonated drinks. There was no difference by weight category. Those taking antipsychotic medication and currently managing their weight were more likely to experience weight gain or fluctuation. ***Conclusions***: These results suggest that excess weight and poor diet quality are a major problem in adults with SMI and that current weight management provision is ineffective in addressing the specific needs of people living with SMI.

## 1. Introduction

Individuals with severe mental illness (SMI) including schizophrenia and bipolar disorder experience poorer physical health than the general population. This group have a reduced life expectancy of 15–25 years [1,2,3,4]. Higher death rates are attributed to chronic physical health conditions including liver disease, respiratory disease, cardiovascular disease and cancer and also the behavioural consequences of living with SMI such as substance abuse and suicide [5].

This mortality gap is due to a number of modifiable and non-modifiable risk factors. Many are linked to the illness itself and associated medication and others are associated with the socio-economic environment in which these individuals live. People living in the most deprived economic areas are twice as likely to experience SMI than those living in the least deprived areas [6]. For example, 23% of men and 26% of women living in the most deprived areas of Scotland reported psychiatric disorder, compared with 12 and 16% of men and women living in the most affluent areas [7].

One area that requires attention in this group is the high levels of overweight and obesity. People with SMI are 1.8 times more likely to live with obesity, that is a body mass index (BMI) ≥ 30 kg/m^2^ (PHE, 2018 [5]). Obesity is concerning as it is linked with other serious long term physical health complications such as diabetes, hypercholesterolaemia, metabolic syndrome, increased cardiovascular and cerebrovascular morbidity and mortality. It is also associated with psychological co-morbidity including mood disorder, reduced quality of life and poor drug compliance [8,9,10,11].

The risk factors for higher rates of obesity in this group are poorly understood. People with SMI have higher rates of poverty and are more likely to live in obesogenic environments. These are characterised by limited access and resource for healthy food and where the built environment is not analogous with physical activity [5,12]. Diet quality is poorer in individuals with SMI and they are more likely to consume cheaper foods that are energy-dense, higher in salt and sugar and consume less fruit and vegetables [8]. In a large survey of adults with SMI, almost 8.5% reported eating no fruit or vegetables [13,14]. Lack of consumption of fruit and vegetables was more common in younger males, those who were unemployed, living in deprived neighbourhoods and those who did not rate healthy living as important [14]. Low food literacy and disordered eating patterns (binge eating, emotional eating and strict dietary restriction) are also common in people with SMI [8,15,16,17].

A unique risk factor for this group in terms of weight management is the routine use of antipsychotic medication and weight change. Throughout this paper we use the umbrella term ‘*weight change*’. This term includes ‘*weight gain*’ which we define as gaining 6 kg or more and ‘*weight fluctuation’* which refers to gaining ≥6 kg more than once. There is an established literature on the relationship between both typical antipsychotics (such as chlorpromazine) and newer atypical antipsychotic medications (clozapine, olanzapine risperidone) and significant and rapid weight gain [9]. However, there is variation in the propensity of these drugs to cause weight gain. The World Health Organisation guidelines recommend clinicians considers psychotropic medication with lower propensity to weight gain for people with SMI who are overweight, with obesity or at risk of developing obesity [18]. However, we know little about the characteristics of people with SMI who are prescribed these medications and their associations with weight change and eating behaviours. With this in mind, we conducted a large cross-sectional survey of adults living with SMI, which aimed to:

1. understand the characteristics of people with SMI in terms of current weight, weight change and diet quality of people with SMI;
2. explore the association between participant weight change and.
  a. diet quality and current weight category;
  b. antipsychotic medication;
  c. current weight management.

## 2. Materials and Methods

### 2.1. Study Design

This was a cross-sectional study in which a survey was completed online, by post, or with a researcher either over the telephone or in person. The study was conducted according to the guidelines laid down in the Declaration of Helsinki and all procedures involving human subjects/patients were approved by the West of Scotland Research Ethics Service in June 2018 (reference 18/WS/0107).

### 2.2. Participants

To be eligible to complete the survey, participants had to be aged 18 years or over and have a documented diagnosis of schizophrenia, schizoaffective disorder or bipolar according to the latest diagnostic criteria at the time of study (DSM5 or ICD-10). The study aimed to recruit 500 participants based on expected response rate and experience from previous research.

### 2.3. Recruitment and Procedures

There were two phases of recruitment for this study, prior to the COVID-19 pandemic (March 2020) and after the onset of the COVID-19 pandemic (from June 2021 until March 2022). The pre-pandemic recruitment method involved mass mailouts to people who had taken part in the Closing the Gap: Health and Wellbeing Cohort (described in Peckham et al., 2023 [12]) and had consented to recontact. Mailouts were sent from five sites, three secondary care NHS mental health trusts and two clinical commissioning groups. Those invited to take part were sent a postal invitation pack containing an invitation letter, participant information sheet the survey and a pre-paid envelope. Those who were interested in taking part were asked to complete and return the survey in the pre-paid envelope provided. After the commencement of the pandemic it became impractical to conduct mass mailouts due to limited access to office space, therefore a mainly remote method of recruitment was adopted when the study recommenced in June 2021. From June 2021 to March 2022 participants were recruited either from research databases held by NHS mental health trusts or through clinical contacts. Those who expressed an interest in taking part in the study were invited, in order of preference, to complete the survey over the phone with a researcher or when the pandemic restrictions were lifted complete the survey face to face with a researcher, complete the survey via on online link sent by SMS message or email, or via a hard copy sent in the post. Voluntary completion of the survey indicated consent to take part in the study. The survey took approximately 20 min to complete and participants maintained anonymity.

### 2.4. Measures

The bespoke survey was developed by a multi-disciplinary research team of researchers and people with lived experience of SMI, a copy of which can be found on the Open Science Framework (https://osf.io/rgc62/, 26 March 2025). The survey had three sections: (A) physical activity and sedentary behaviour; (B) health; (C) socio-demographics. An overview of the measures included in this article are provided below. Analyses of physical activity and sedentary behaviour are reported elsewhere [19]. Minor amendments were made to the survey questions prior to the second wave of recruitment, PAQ1 refers to the initial version and PAQ2 the later version.

#### 2.4.1. Health Measures

Participants were asked to rate their mental health in last 12 months as excellent, good, moderate, poor or very poor. Smoking status was recorded via the question ‘do you smoke?’ with responses; yes, no, I have never smoked, and no, but I used to smoke. Participants were then asked to rate their average level of fatigue in the last two weeks along with how much of their waking time they have spent feeling fatigued in the last two weeks, on a 5-point likert scale. The scores for each domain were multiplied to give a composite score of fatigue. Depression severity was measured using the Patient Health Questionnaire-8 (PHQ-8) [20]. The PHQ-8 is an 8-item depression measure designed for population-based research. It omits the suicide question from the longer PHQ-9 [21], which is deemed unsuitable for remote administration. Items are scored between 0 = not at all to 3 = nearly everyday, resulting in a total score of 0–24. A cut-off of ≥10 has a sensitivity and specificity of 88% for detecting major depression.

BMI was calculated via self-reported height and weight with a BMI of less than 18.5 being coded as underweight, 18.5–24.9 being healthy weight, 25–29.9 being overweight and 30 or having obesity. Self-reported lowest ever adult weight and highest ever adult weight and weight fluctuation were also recorded. To explore weight management participants were asked whether they were currently managing their weight and if so, i. whether it was to lose weight, maintain weight or gain weight and ii. how they were managing their weight (diet, exercise, other).

Diet was assessed through asking people how many portions of fruit and vegetables they eat per typical day, with options of 1, 2, 3, 4 or 5 or more. In addition, the number of carbonated beverages they consumed per day, with options of 1, 2, 3, 4, or 5 or more.

Participants were asked to list any medication they were taking with the option to leave that section blank if they didn’t wish to say what medication they were taking. The complete list of medications reported was reviewed by the National Specialty Advisor Mental Health Pharmacy for NHS England (PP), who categorised them as being antipsychotic medication or non-antipsychotic medication. Antipsychotic medications were then categorised as low, medium or high propensity to weight gain based on findings of the Lancet review by Huhn et al. of 402 studies and 53,463 participants [22].

#### 2.4.2. Sociodemographic Measures

Information was collected on age, gender and ethnicity.

### 2.5. Data Analysis

All analyses were performed using Stata 17 software (StataCorp, 2021. Stata Statistical Software: Release 17. College Station, TX, USA: StataCorp LLC.). Descriptive statistics (e.g., % [n], mean [SD], median [IQR]) were used to summarise respondent characteristics for the full cohort and by BMI classification.

Measures of weight change (weight gain, weight fluctuation, weight management) were compared between participants that reported using antipsychotic medication and those that did not via univariable and multivariable (adjusted for age and sex) logistic regression. Odds ratios and corresponding 95% confidence intervals were calculated. Participants who did not report any medication use were excluded from these analyses.

Weight gain and weight fluctuation were compared between participants who reported currently managing weight and those who reported not currently managing weight using logistic regression analogous to the above methods. Weight change (difference between lowest and highest adult weight) was compared between participants managing their weight and not managing their weight using univariable and multivariable (adjusted for age and sex) linear regression. Mean differences and corresponding 95% confidence intervals were calculated.

All analyses were performed using available data, characteristics were descriptively compared between participants that reported their medication use and those that did not (See https://osf.io/rgc62/ for PAQ analysis plan).

## 3. Results

### 3.1. Participant Characteristics

Five hundred and twenty nine people completed the survey, 196 completed a postal survey, 106 people completed the survey online and 186 people completed the survey over the phone or in person (41). One hundred and fifty five completed PAQ1 and 374 people completed PAQ2 (the later version). Participant demographics and characteristics are detailed in Table 1.

The association between diet quality, weight management and mental wellbeing characteristics are presented by BMI classification in Table 2.

### 3.2. Association Between Medication Use and Weight Change

Seventy six percent (n = 403/529) of participants reported their medication use. The characteristics of the participants that reported medication use are compared to those that did not report medication use in Supplementary Material Table S1. Participants who reported their medication use were more likely to be male and professionally active.

Table 3 presents associations between antipsychotic medication use and weight change for the participants that reported any medication use (N = 403). The odds of weight gain (gaining >6 kg and not losing again) and weight fluctuation (weight gain more than once) were slightly increased in the participants who reported taking antipsychotics (weight gain was reported in 51% of antipsychotic users and 41% of non users, adjusted OR 1.31), but the confidence intervals for both unadjusted and adjusted analyses did not exclude a small decrease. Participants current weight management (yes/no) was not associated with antipsychotic medication use (adjusted OR for managing weight; 0.90, 95% CI 0.53 to 1.53).

### 3.3. Association Between Attempts at Weight Management and Weight Change

Table 4 presents associations between current weight management and adult weight gain. Weight gain (gaining >6 kg and not losing it again) was reported by 47% of the participants currently managing their weight and 43% of the participants not currently managing their weight. The odds of having gained were higher in the participants currently managing their weight (adjusted OR 1.32) but the confidence interval was not conclusive. Weight fluctuation was reported in 17% of the participants currently managing their weight and 11% of the participants not managing their weight. Weight management was associated with higher odds of weight fluctuation (adjusted OR 1.73, 95% CI 1.00 to 2.96).

Weight change (difference between lowest and highest adult weight) was similar in the group that were currently managing weight and not managing weight (35.1 kg compared to 33.3 kg). After adjustment for age and sex, the mean difference between the two group was estimated to be 2.36 kg (i.e., weight change is 2.36 kg larger in the managing weight group), the confidence interval for this estimate was large (95% CI −2.14 to 6.86) lifetime weight change was not associated with current weight management.

## 4. Discussion

The current study presents the findings of a large cross-sectional survey of adults living with SMI. The survey aimed to understand the current weight, weight change and diet quality of this group and explore the factors associated with weight change.

Levels of obesity were notably higher in the current sample (42%) than in our earlier cohort of adults with SMI (37.5%) [14] and also compared to the general population (28%) [23]. A mean BMI of 31.2 kg/m^2^ puts the sample overall in the ‘obese’ range. Diet quality was also evidently poor. Only 17% of our sample met the recommended five portions of fruit and vegetables a day and 6% reported consuming no fruit or vegetables. This is lower than in the general population (where 25% report meeting the recommendations) [24].

Results showed there was no association between weight category and diet quality in this cohort. Meaning that those of higher weight did not consume less fruit and vegetables or drink more carbonated drinks as suggested in the literature. This suggests that excess weight may not be a result of poor diet alone, but may be associated with other factors related to living with SMI such as the side effects of antipsychotic medication, lack of physical activity and/or food poverty. In line with this, [18] showed that those of a healthy weight were more likely to be sufficiently active compared to those living with obesity. Similarly, our current findings indicated that individuals living with obesity were more likely to be those taking antipsychotic medication and to have experienced weight gain and weight fluctuation.

Almost two-thirds (62%) of our sample reported currently managing their weight and this did not differ across weight categories. This is substantially higher than observed in the general population. A large global review showed prevalence rates of 42% and this increased with the prevalence of overweight and obesity [25]. Similarly, recent trends in England show 30% of healthy weight adults are actively trying to lose weight and this increases with overweight (53%) and obesity (76%) [26]. The findings in our cohort may be due to a sense of hopelessness about weight. People with SMI have told us it feels pointless trying to manage weight while taking weight inducing medication. Furthermore, restricting our questions to ‘current weight management’ may have overlooked multiple past attempts. Future research may want to look at people’s optimism regarding their future success in weight management.

In the current study, individuals currently managing their weight were those who were more likely to have experienced weight fluctuation. Weight cycling as seen in this cohort can be serious and has been associated with greater risk of mortality, chronic diseases and depression [27]. There is a body of evidence developing around disordered eating in patients with SMI. Binge eating disorder, emotional eating and reactive eating patterns are commonly observed in people with schizophrenia and bipolar [8,15,16,17] with less than half of people receiving support [25]. This said, PHQ scores analysed by weight category, did not indicate emotional eating therefore a specific measure of eating behaviour would have been beneficial.

The high levels of overweight and obesity in this sample along with the high proportion who report actively managing their weight, suggests that current weight management options are inadequate in addressing the specific needs of people with SMI, namely medication management. Studies have shown that people with SMI may also require more regular contact, with tailored materials and support [26]. A recent systematic review highlighted both the lack of effective weight management interventions and understanding of the unique barriers for people with SMI accessing these [27]. Behavioural approaches in isolation have largely been unsuccessful and without consideration of the impact of antipsychotic medication, they only address part of the complex issue.

## 5. Strengths and Limitations

The major strength of this study is the large sample, recruited from a range of localities and clinical settings across the UK, which increases the generalisability of our findings. However, the study was cross-sectional in nature so we were unable to infer causality or the temporal relationship between food consumption, medication use, weight management and weight change. Furthermore, the survey relied on self-report therefore, whilst data was reasonably complete, accurate recall of medication use and food consumption can be poor. People living with SMI can require additional support in completing self-report measures, therefore we offered several options for completion to optimize data quality including; telephone support, face to face completion, online or paper. Height and weight were the most commonly missing metrics, which meant we were unable to calculate BMI for 92 participants.

We used fruit and vegetable and carbonated drink consumption as a proxy for diet quality however, a more complete assessment of diet quality such as diary methods in a small subsample of participants would be beneficial in future studies. In this large-scale survey, we tried to ensure that the questions relating to diet were simple for people to understand and complete in case they were completing the survey unaided. As a result of the autonomy needed to complete the survey, the sample did not include inpatients, so may not reflect the more severe presentations and those on higher doses of antipsychotic medication. Finally, in line with the research aims, we analysed antipsychotic medication versus other medication, but further consideration of health conditions associated with weight gain may be warranted.

## 6. Conclusions

High levels of obesity are observed in adults with SMI. While diet quality is poor, it is not linked to weight status, suggesting other factors such as antipsychotic medication may be associated with excess weight in this group. Two thirds of these individuals were actively managing their weight, which demonstrates that people with SMI are concerned about their weight and weight gain, however current weight management provision for adults with SMI is inadequate. Further work needs to be done to develop tailored interventions for this group that take into account the side effects of antipsychotic medication.

## Figures and Tables

**Table 1:** Demographics and characteristics of N = 529 survey respondents.

Characteristic		n (%)
Age (years)	Number (%) of responses	519 (98%)
	Mean (SD)	49.3 (13.1)
	Min., Max.	20, 86
Gender	Female	212 (40%)
	Male	308 (58%)
	Prefer not to say	4 (1%)
	Transgender	3 (1%)
	Missing	2 (0%)
Weight & Weight management	BMI (kg/m^2^) Mean (SD)	31.2 (7.8)
	underweight (<18.5)	2 (0%)
	healthy weight (18.5–24.9)	93 (18%)
	overweight (25–29.9)	119 (22%)
	obese (≥30)	223 (42%)
	Missing	92 (17%)
	Lowest adult weight (kg)	
	Number (%) of responses	421 (80%)
	Mean (SD)	65.5 (17.9)
	Min., Max.	31, 174
	Highest adult weight (kg)	
	Number (%) of responses	427 (81%)
	Mean (SD)	99.1 (26.8)
	Min., Max.	44, 250
	Difference between lowest and highest adult weight (kg)	
	Number (%) of responses	399 (75%)
	Mean (SD)	34.4 (21.7)
	Min., Max.	0, 158
	Currently managing weight	327 (62%)
	Managing weight via diet	168 (32%)
	Managing weight via exercise	139 (26%)
	Reason for managing weight	
	Gain weight	12 (2%)
	Lose weight	223 (42%)
	Stay the same weight	92 (17%)
	Missing	202 (38%)
	Gained weight (>6 kg)	
	No	267 (50%)
	Yes, once	160 (30%)
	Yes, more than once (fluctuation)	80 (15%)
	Missing	22 (4%)
Employment	Professionally active	99 (19%)
	Not professionally active	356 (67%)
	Other	21 (4%)
	Missing	53 (10%)
Ethnicity	UK/Irish/other white	416 (79%)
	Asian	21 (4%)
	Black/African/Caribbean	26 (5%)
	Mixed multiple ethnic groups	14 (3%)
	Other	12 (2%)
	Missing	40 (8%)
Diet quality	Consumption of fruit and vegetables	
	0	32 (6%)
	1	102 (19%)
	2	119 (22%)
	3	122 (23%)
	4	58 (11%)
	5 or more	91 (17%)
	Missing	5 (1%)
	Carbonated drinks consumption	
	I do not drink carbonated soft drinks	194 (37%)
	Less than one time per day	113 (21%)
	1 time per day	71 (13%)
	2 times per day	45 (9%)
	3 times per day	40 (8%)
	4 times per day	21 (4%)
	5 times per day	34 (6%)
	Missing	11 (2%)
Medication	Reported any medication use	403 (76%)
	Reported taking antipsychotics	318 (60%)
	Medicine weight gain propensity	
	Low	83 (16%)
	Medium	59 (11%)
	High	151 (29%)
	Missing	236 (45%)
Depression	PHQ-8 Depression severity	
	0–4 (minimal)	159 (30%)
	5–9 (mild)	120 (23%)
	10–14 (moderate)	85 (16%)
	15–19 (moderately severe)	72 (14%)
	20–24 (severe)	47 (9%)
	Missing	46 (9%)

**Table 2:** Participant characteristics by BMI classification (n = 435).

Characteristic	Healthy Weight	Overweight	Obese
	(n = 93)	(n = 119)	(n = 223)
Consumption of fruit and vegetables			
four or less portions a day	81 (87%)	97 (82%)	174 (78%)
Five or more portions a day	11 (12%)	21 (18%)	48 (22%)
Carbonated drinks consumption			
I do not drink carbonated soft drinks	35 (38%)	50 (42%)	84 (38%)
less than one time per day	24 (26%)	23 (19%)	46 (21%)
1 time per day	14 (15%)	18 (15%)	21 (9%)
2 times per day	7 (8%)	9 (8%)	21 (9%)
3 times per day	7 (8%)	5 (4%)	25 (11%)
4 times per day	2 (8%)	6 (5%)	7 (3%)
5 times per day	2 (8%)	6 (5%)	17 (8%)
Medicine weight gain propensity			
low	10 (11%)	26 (22%)	41 (18%)
medium	17 (18%)	9 (8%)	27 (12%)
high	25 (27%)	39 (33%)	69 (31%)
Reason for managing weight			
gain weight	5 (5%)	1 (1%)	1 (0%)
lose weight	23 (25%)	48 (40%)	122 (55%)
stay the same weight	28 (30%)	27 (23%)	21 (9%)
Gained weight (>6 kg)			
No	69 (74%)	66 (56%)	79 (35%)
Yes, once	17 (18%)	36 (30%)	83 (37%)
Yes, more than once	4 (4%)	14 (12%)	55 (25%)
Reported any medication use	80 (86%)	98 (82%)	185 (83%)
Reported taking antipsychotics	55 (59%)	78 (66%)	153 (69%)
Currently managing weight	60 (65%)	77 (65%)	140 (63%)
Managing weight via diet	26 (28%)	38 (32%)	87 (39%)
Managing weight via exercise	22 (24%)	35 (29%)	62 (28%)
PHQ-8 depression severity			
0–4 (minimal depression)	34 (37%)	37 (31%)	63 (28%)
5–9 (mild depression)	18 (19%)	33 (28%)	48 (22%)
10–14 (moderate depression)	11 (12%)	20 (17%)	36 (16%)
15–19 (moderately severe depression)	9 (10%)	18 (15%)	31 (14%)
20–24 (severe depression)	13 (14%)	3 (3%)	25 (11%)

NB: data is not presented for two underweight participants and 92 participants whose BMI couldn’t be calculated due to missing height or weight data.

**Table 3:** Association between current antipsychotic medication use and weight change for participants that reported medication use (N = 403).

Weight Change		Uses Antipsychotic Medication	Does Not Use Antipsychotic Medication	Unadjusted ^a^		Adjusted ^b^	
		(N = 318)	(N = 85)				
		n (%)	n (%)	OR	95% CI	OR	95% CI
Weight gain	No	146 (46%)	48 (56%)				
	Yes	162 (51%)	35 (41%)	1.52	0.93 to 2.48	1.31	0.78 to 2.20
Weight fluctuation	No	250 (79%)	71 (84%)				
	Yes	58 (18%)	12 (14%)	1.37	0.70 to 2.70	1.62	0.80 to 3.25
Weight management	No	118 (37%)	27 (32%)				
	Yes	198 (62%)	54 (64%)	0.84	0.50 to 1.40	0.90	0.53 to 1.53

(a) Unadjusted based on logistic regression of weight measure against antipsychotic medication use (dependent variable), (b) adjusted based on logistic regression of weight measure against antipsychotic medication use (dependent variable), age and sex (simplified to be male vs. female, prefer not to say and transgender included in male category due to small numbers). CI confidence interval. OR (Odds ratio); odds of weight measure for those using antipsychotic medication compared to those not using antipsychotic medication.

**Table 4:** Association between current weight management and weight gain/fluctuation/change (N = 520).

		Managing Weight	Not Managing Weight	Unadjusted		Adjusted	
		(N = 327)	(N = 193)				
Weight measure		n (%)	n (%)	OR ^a^	95% CI	OR ^b^	95% CI
Weight gain	No	162 (50%)	103 (53%)				
	Yes	155 (47%)	83 (43%)	1.19	0.83 to 1.71	1.32	0.91 to 1.93
Weight fluctuation	No	260 (80%)	164 (85%)				
	Yes	57 (17%)	22 (11%)	1.63	0.96 to 2.77	1.73	1.00 to 2.96
				Mean difference ^c^	95% CI	Mean difference ^d^	95% CI
Weight change (Difference between lowest and highest adult weight (kg))	N (%)	254 (78%)	142 (74%)				
	Mean (SD)	35.1 (21.5)	33.3 (22.2)	1.86	−2.62 to 6.35	2.36	−2.14 to 6.86

Odds ratio (OR) odds of weight gain/weight fluctuation for participants that are currently managing their weight compared to those that aren’t based on (a) (unadjusted) logistic regression, (b) adjusted OR based on logistic regression with covariates of age and sex. Mean difference in weight change between participants that are currently managing their weight compared to those that aren’t, based on (c) unadjusted linear regression and (d) linear regression adjusted for age and sex.

## Data Availability

The anonymized dataset may be made available on request from author EP.

## Supplementary Materials

The following supporting information can be downloaded at: https://www.mdpi.com/article/10.3390/nu17091423/s1, Table S1: Participant descriptive by medication reporting status.

## Abbreviations

The following abbreviations are used in this manuscript:

BMI	Body mass index
DSM5	Diagnostic and Statistical Manual version 5
ICD-10	International Classification of Disease version 10
Kg	Kilogram
NHS	National Health Service
PAQ	Physical Activity Questionnaire
PHQ	Patient Health Questionnaire
SMI	Severe mental illness
