# Supplementary material for: Factors Associated with Weight Change in Adults with Severe Mental Illness: Results from a Large Cross-Sectional Survey
Source: Nutrients. 2025 Apr 23;17(9):1423. doi: 10.3390/nu17091423 (PMC12073341; doi:10.3390/nu17091423)
Supplement: Supplementary file 1 [file nutrients-17-01423-s001.zip › nutrients-3582123-supplementary.pdf]

Table S1. Participant descriptive by medication reporting status

Characteristic		Reported medication	Did not report medication	All
		(n=403)	(n=126)	(n=529)
Age (years)				
	N (%)	399 (99%)	120 (95%)	519 (98%)
	Mean (SD)	49 (13)	49 (14)	49 (13)
	Median (IQR)	49 (41, 59)	49 (40, 59)	49 (41, 59)
BMI (kg/m2)				
	N (%)	364 (90%)	73 (58%)	437 (83%)
	Mean (SD)	31 (8)	31 (7)	31 (8)
	Median (IQR)	30 (26, 35)	30 (27, 34)	30 (26, 35)
Age (years)				
	18-31	60 (15%)	21 (17%)	81 (15%)
	35-64	288 (71%)	83 (66%)	371 (70%)
	>=65	51 (13%)	16 (13%)	67 (13%)
BMI (kg/m2)				
	underweight (<18.5)	1 (0%)	1 (1%)	2 (0%)
	healthy weight (18.5-24.9)	80 (20%)	13 (10%)	93 (18%)
	overweight (25-29.9)	98 (24%)	21 (17%)	119 (22%)
	obese (>=30)	185 (46%)	38 (30%)	223 (42%)
Survey group				
	PAQ1	126 (31%)	29 (23%)	155 (29%)
	PAQ2	277 (69%)	97 (77%)	374 (71%)
Gender				
	Female	155 (38%)	57 (45%)	212 (40%)
	Male	242 (60%)	66 (52%)	308 (58%)
	Prefer not to say	4 (1%)	0 (0%)	4 (1%)
	Transgender	2 (0%)	1 (1%)	3 (1%)
Employment				
	Professionally active	87 (22%)	12 (10%)	99 (19%)
	Not professionally active	290 (72%)	66 (52%)	356 (67%)
	Other	15 (4%)	6 (5%)	21 (4%)
Ethnicity				
	UK/Irish/other white	346 (86%)	70 (56%)	416 (79%)
	Asian	19 (5%)	2 (2%)	21 (4%)
	Black/African/Caribbean	17 (4%)	9 (7%)	26 (5%)
	Mixed multiple ethnic groups	9 (2%)	5 (4%)	14 (3%)
	Other	10 (2%)	2 (2%)	12 (2%)
Self rated mental health				
	Excellent	37 (9%)	13 (10%)	50 (9%)

Good	115 (29%)	34 (27%)	149 (28%)
Moderate	126 (31%)	40 (32%)	166 (31%)
Poor	71 (18%)	28 (22%)	99 (19%)
Very poor	49 (12%)	9 (7%)	58 (11%)
PHQ-8 depression severity			
0-4 (minimal depression)	116 (29%)	43 (34%)	159 (30%)
5-9 (mild depression)	99 (25%)	21 (17%)	120 (23%)
10-14 (moderate depression)	66 (16%)	19 (15%)	85 (16%)
15-19 (moderately severe depression)	56 (14%)	16 (13%)	72 (14%)
20-24 (severe depression)	38 (9%)	9 (7%)	47 (9%)
Smoking			
No, I have never smoked	133 (33%)	38 (30%)	171 (32%)
No, but I used to smoke	130 (32%)	36 (29%)	166 (31%)
Yes	134 (33%)	51 (40%)	185 (35%)
Consumption of fruit and vegetables			
four or less portions a day	323 (80%)	110 (87%)	433 (82%)
Five or more portions a day	77 (19%)	14 (11%)	91 (17%)
Fatigue score			
0 (none)	53 (13%)	18 (14%)	71 (13%)
1-10 (slight to moderate)	276 (68%)	83 (66%)	359 (68%)
11-20 (severe)	59 (15%)	14 (11%)	73 (14%)
